# Supplementary material for: Predicting brain volumes from anthropometric and demographic features: insights from UK biobank neuroimaging data
Source: Brain Struct Funct. 2026 Mar 11;231(3):37. doi: 10.1007/s00429-025-03070-9 (PMC12979296; doi:10.1007/s00429-025-03070-9)

**Supplementary Material**

**List of tables and figures**

| **Table S1** | Definition of the healthy controls (HC) based on ICD-10 exclusion criteria |
| --- | --- |
| **Table S2** | Comparison of linear support vector machine (SVM) and Random Forest (RF) model performance based on corrected repeated k-fold paired t-tests for across-sex analyses. Reported values show the mean difference in Pearson’s r (SVM − RF), corresponding t-values, and p-values for each brain volume measure and preprocessing pipeline (CAT and FreeSurfer). |
| **Table S3** | Comparison of linear support vector machine (SVM) and Random Forest (RF) models on the training dataset for all brain volumes |
| **Table S4** | Comparison between ANOVA and ML (Haufe transform) results for TIV and TBV |
| **Figure S1** | Prediction of head size: Total intracranial volume (TIV) on CAT data using RF. |
| **Figure S2** | Prediction of head size: Total intracranial volume (TIV) on FreeSurfer data using linear SVM. |
| **Figure S3** | Prediction of head size: Total intracranial volume (TIV) on FreeSurfer data using RF. |
| **Figure S4** | Prediction of brain size: Total brain volume (TBV) on CAT data using RF. |
| **Figure S5** | Prediction of brain size: Total brain volume (TBV) on FreeSurfer data using linear SVM. |
| **Figure S6** | Prediction of brain size: Total brain volume (TBV) on FreeSurfer data using RF. |
| **Figure S7** | Impact of age on brain volumes for across-sex analysis on CAT data using RF. |
| **Figure S8** | Impact of age on brain volumes for within-sex analysis on CAT data using linear SVM. |
| **Figure S9** | Impact of age on brain volumes for within -sex analysis on CAT data using RF. |
| **Figure S10** | Impact of age on brain volumes for across-sex analysis on FreeSurfer data using linear SVM. |
| **Figure S11** | Impact of age on brain volumes for across -sex analysis on FreeSurfer data using RF. |
| **Figure S12** | Impact of age on brain volumes for within-sex analysis on FreeSurfer data using linear SVM. |
| **Figure S13** | Impact of age on brain volumes for within-sex analysis on FreeSurfer data using RF. |

**Table S1:** Definition of the healthy controls (HC) based on ICD-10 exclusion criteria

| Populations | Excluded ICD-10 criteria |
| --- | --- |
| Healthy controls (HC) | Mental and behavioural disorders: F |
|  | Diseases of the nervous system: G |
|  | Cerebrovascular diseases: I60-I69 |
|  | Diseases of the musculoskeletal system and connective tissue: M |
|  | Injury, poisoning, and certain other consequences of external causes: S |

**Table S2:** Comparison of linear support vector machine (SVM) and Random Forest (RF) model performance based on corrected repeated k-fold paired t-tests for across-sex analyses. Reported values show the mean difference in Pearson’s r (SVM − RF), corresponding t-values, and p-values for each brain volume measure and preprocessing pipeline (CAT and FreeSurfer).

| Dataset | Brain volume | Mean diff (SVM − RF) | t -value | p-value |
| --- | --- | --- | --- | --- |
| CAT | TIV | 0.031 | 11.57 | 0.00 |
|  | TBV | 0.033 | 11.7 | 0.00 |
|  | GMV | 0.037 | 12.24 | 0.00 |
|  | WMV | 0.036 | 11.53 | 0.00 |
|  | CFS | 0.026 | 9.1 | 9.76 |
| FreeSurfer | TIV | 0.035 | 11.27 | 0.0 |
|  | TBV | 0.035 | 11.25 | 0.0 |
|  | GMV | 0.037 | 12.27 | 0.0 |
|  | WMV | 0.035 | 11.78 | 0.0 |
|  | CFS | 0.062 | 10.9 | 0.0 |

**Table S3:** Comparison of linear support vector machine (SVM) and Random Forest (RF) models on the training dataset for all brain volumes in CAT and FreeSurfer data.

|  |  |  |  | Linear SVM | | |  | RF | | |
| --- | --- | --- | --- | --- | --- | --- | --- | --- | --- | --- |
| Data | Brain volume | Scores  (mean±SD) |  | Both sexes | Female | Male |  | Both sexes | Female | Male |
| CAT | TIV | Pearson’s r |  | 0.67±0.01 | 0.24±0.03 | 0.24±0.03 |  | 0.63±0.01 | 0.16±0.01 | 0.16±0.03 |
|  |  | R2 |  | 0.44±0.01 | 0.06±0.01 | 0.06±0.02 |  | 0.4±0.02 | -0.03±0.02 | -0.02±0.02 |
|  |  | ﻿MAE |  | 88.03±1.35 | 82.18±1.67 | 94.81±2.49 |  | 91.64±1.64 | 85.66±1.76 | 98.2±2.52 |
|  | TBV | Pearson’s r |  | 0.64±0.01 | 0.39±0.02 | 0.43±0.02 |  | 0.61±0.01 | 0.33±0.02 | 0.38±0.02 |
|  |  | R2 |  | 0.41±0.01 | 0.15±0.02 | 0.19±0.02 |  | 0.36±0.02 | 0.08±0.02 | 0.12±0.03 |
|  |  | ﻿MAE |  | 70.57±1.17 | 66.02±1.31 | 75.54±1.92 |  | 73.46±1.25 | 68.77±1.4 | 78.49±2.00 |
|  | GMV | Pearson’s r |  | 0.62±0.01 | 0.41±0.02 | 0.45±0.02 |  | 0.58±0.01 | 0.35±0.02 | 0.39±0.03 |
|  |  | R2 |  | 0.38±0.01 | 0.17±0.02 | 0.2±0.02 |  | 0.33±0.02 | 0.1±0.02 | 0.13±0.03 |
|  |  | ﻿MAE |  | 36.37±0.59 | 34. 4±0.74 | 38.51±0.99 |  | 37.91±0.68 | 35.82±0.77 | 40.18±1.04 |
|  | WMV | Pearson’s r |  | 0.6±0.01 | 0.33± 0.02 | 0.37±0.02 |  | 0.57±0.01 | 0.25±0.02 | 0.31±0.03 |
|  |  | R2 |  | 0.36±0.02 | 0.11± 0.02 | 0.13±0.02 |  | 0.31±0.02 | 0.03±0.02 | 0.07±0.03 |
|  |  | ﻿MAE |  | 40.19±0.7 | 37.33±0.81 | 43.37±1.04 |  | 41.73±0.7 | 38.95±0.86 | 44.76±1.12 |
|  | CSF | Pearson’s r |  | 0.67±0.01 | 0.42±0.03 | 0.58±0.02 |  | 0.64±0.01 | 0.36±0.02 | 0.53±0.02 |
|  |  | R2 |  | 0.44±0.02 | 0.18±0.02 | 0.33±0.02 |  | 0.41±0.01 | 0.11±0.03 | 0.27±0.03 |
|  |  | ﻿MAE |  | 38.2±0.72 | 36.72±0.87 | 39.16±1.00 |  | 39.34±0.74 | 38.23±0.84 | 40.66±1.04 |
| FreeSurfer | TIV | Pearson’s r |  | 0.62±0.01 | 0.29±0.03 | 0.25±0.03 |  | 0.58±0.01 | 0.22±0.03 | 0.18±0.03 |
|  |  | R2 |  | 0.38±0.02 | 0.08±0.02 | 0.06±0.02 |  | 0.33±0.02 | 0.01±0.02 | -0.01±0.02 |
|  |  | ﻿MAE |  | 94998.12±1520.2 | 87784.31±1761.5 | 102807.07±2597.9 |  | 98723.49±1742.2 | 91424.78±1850.1 | 106688.8±2688.2 |
|  | TBV | Pearson’s r |  | 0.64±0.01 | 0.42±0.02 | 0.4±0.02 |  | 0.6±0.01 | 0.35±0.02 | 0.35±0.02 |
|  |  | R2 |  | 0.4±0.02 | 0.17±0.02 | 0.16±0.02 |  | 0.35±0.02 | 0.1±0.02 | 0.1±0.03 |
|  |  | ﻿MAE |  | 68665.65±1113.1 | 64855.27±1321.5 | 72899.48±1747.6 |  | 71480.78±1246.6 | 603.42±1382.95 | 75597.82±1811.14 |
|  | GMV | Pearson’s r |  | 0.62±0.01 | 0.49±0.02 | 0.48±0.02 |  | 0.58±0.01 | 0.44±0.02 | 0.43±0.02 |
|  |  | R2 |  | 0.38±0.01 | 0.24±0.02 | 0.23±0.02 |  | 0.33±0.02 | 0.18±0.03 | 0.16±0.03 |
|  |  | ﻿MAE |  | 34659.68±624.34 | 32935.11±765.31 | 36524.7±959.51 |  | 36134.77±659.67 | 34342.94±814.04 | 38018.93±942.19 |
|  | WMV | Pearson’s r |  | 0.62±0.01 | 0.3±0.03 | 0.3±0.03 |  | 0.58±0.01 | 0.22±0.03 | 0.24±0.03 |
|  |  | R2 |  | 0.38±0.02 | 0.09±0.02 | 0.09±0.02 |  | 0.33±0.02 | 0.01±0.02 | 0.03±0.02 |
|  |  | ﻿MAE |  | 38774.96±654.91 | 36223.49±782.87 | 1639.23±939.53 |  | 40291.96±665.98 | 37826.63±869.79 | 42976.85±979.98 |
|  | CSF | Pearson’s r |  | 0.43±0.02 | 0.23±0.03 | 0.24±0.03 |  | 0.36±0.02 | 0.14±0.03 | 0.16±0.03 |
|  |  | R2 |  | 0.18±0.01 | 0.05±0.01 | 0.05±0.02 |  | 0.11±0.02 | -0.03±0.02 | -0.03±0.02 |
|  |  | ﻿MAE |  | 182.61±3.43 | 168.71±4.03 | 198.39±5.18 |  | 191.4±3.44 | 177.14±4.26 | 207.71±5.32 |

**Table S4:** Comparison Between ANOVA and ML (Haufe transform) Results for TIV and TBV

| Brain volume | Features | ANOVA  F-value | Haufe transform | Interpretation |
| --- | --- | --- | --- | --- |
| **TIV** | **Sex** | **2680.85** | 0.96 | Strongest predictor; reflects clear sex difference in head size, consistent direction. |
|  | **Age** | 11.65 | 0.07 | Very small, negative effect TIV largely stable across age; matches low FI. |
|  | **Weight** | 130.23 | 0.57 | Moderate positive effect; heavier individuals have larger brain volume. |
|  | **WC** | 60.06 | 0.45 | Small positive; weak but consistent with ML direction. |
|  | **HC** | 38.97 | 0.04 | Weak effect; consistent positive sign. |
|  | **Seated height** | 498.64 | 0.73 | Strong positive effect; second only to sex, consistent with ML pattern. |
|  | **Box height** | 272.97 | 0.38 | Moderate positive contribution; same direction as ML. |
| **TBV** | **Sex** | 1721.28 | 0.83 | Strong overall predictor; reflects sex-related difference in brain size but smaller than for TIV. |
|  | **Age** | 1517.14 | -0.34 | Strong negative effect; confirms substantial age-related decline in brain volume. |
|  | **Weight** | 131.94 | 0.57 | Moderate positive effect; larger body size associated with greater brain volume. |
|  | **WC** | 86.13 | 0.37 | Small positive effect; weaker and more variable association across sexes. |
|  | **HC** | 19.26 | 0.07 | Minimal positive effect; consistent direction with ML findings. |
|  | **Seated height** | 541.03 | 0.76 | Strong positive effect; taller individuals tend to have larger brain volumes. |
|  | **Box height** | 282.43 | 0.39 | Moderate positive effect; aligns with anthropometric scaling trends. |

**Figure S1:** Prediction of head size: Total intracranial volume (TIV) on CAT data using RF.


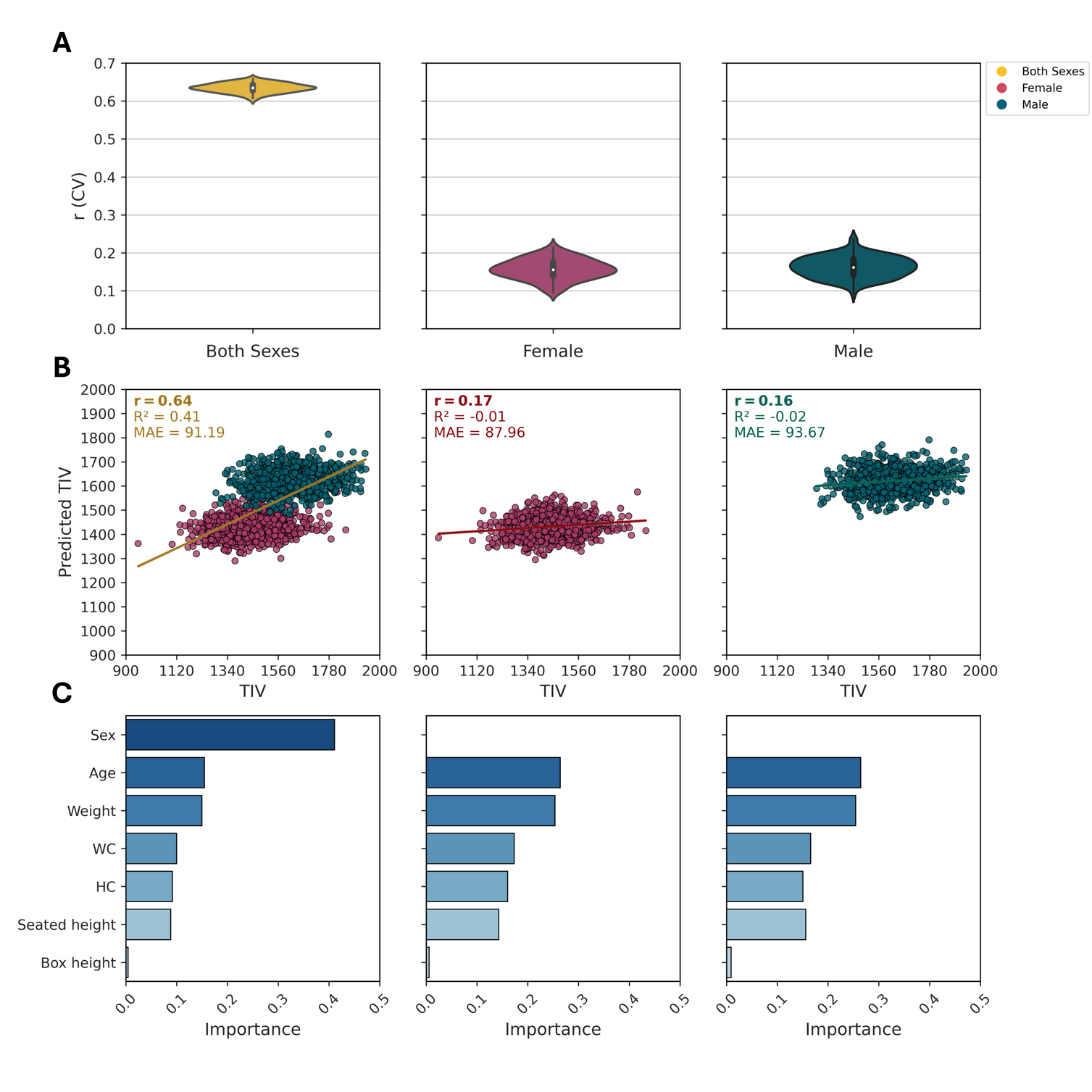


**Figure S2:** Prediction of head size: Total intracranial volume (TIV) on FreeSurfer data using linear SVM.


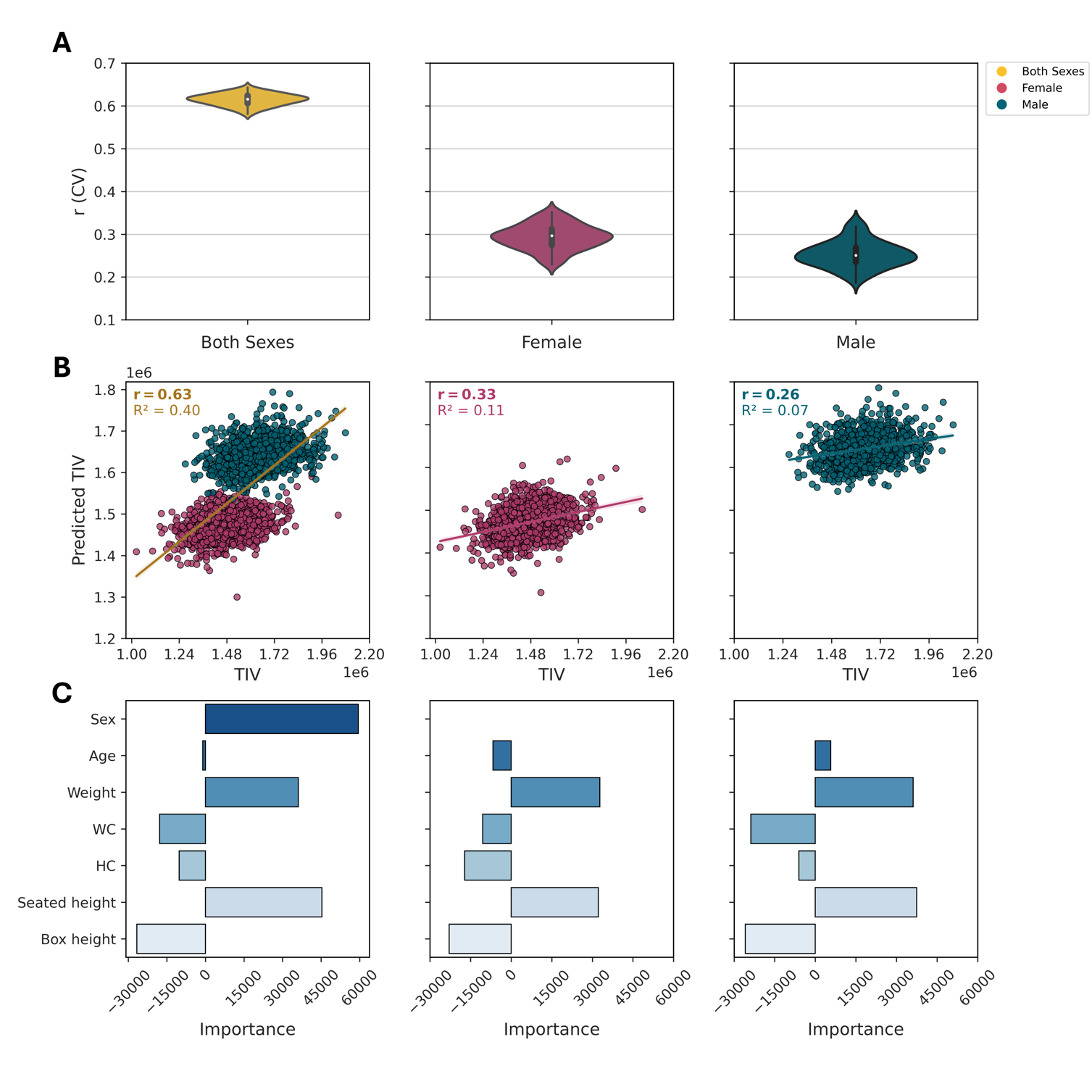


**Figure S3:** Prediction of head size: Total intracranial volume (TIV) on FreeSurfer data using RF.


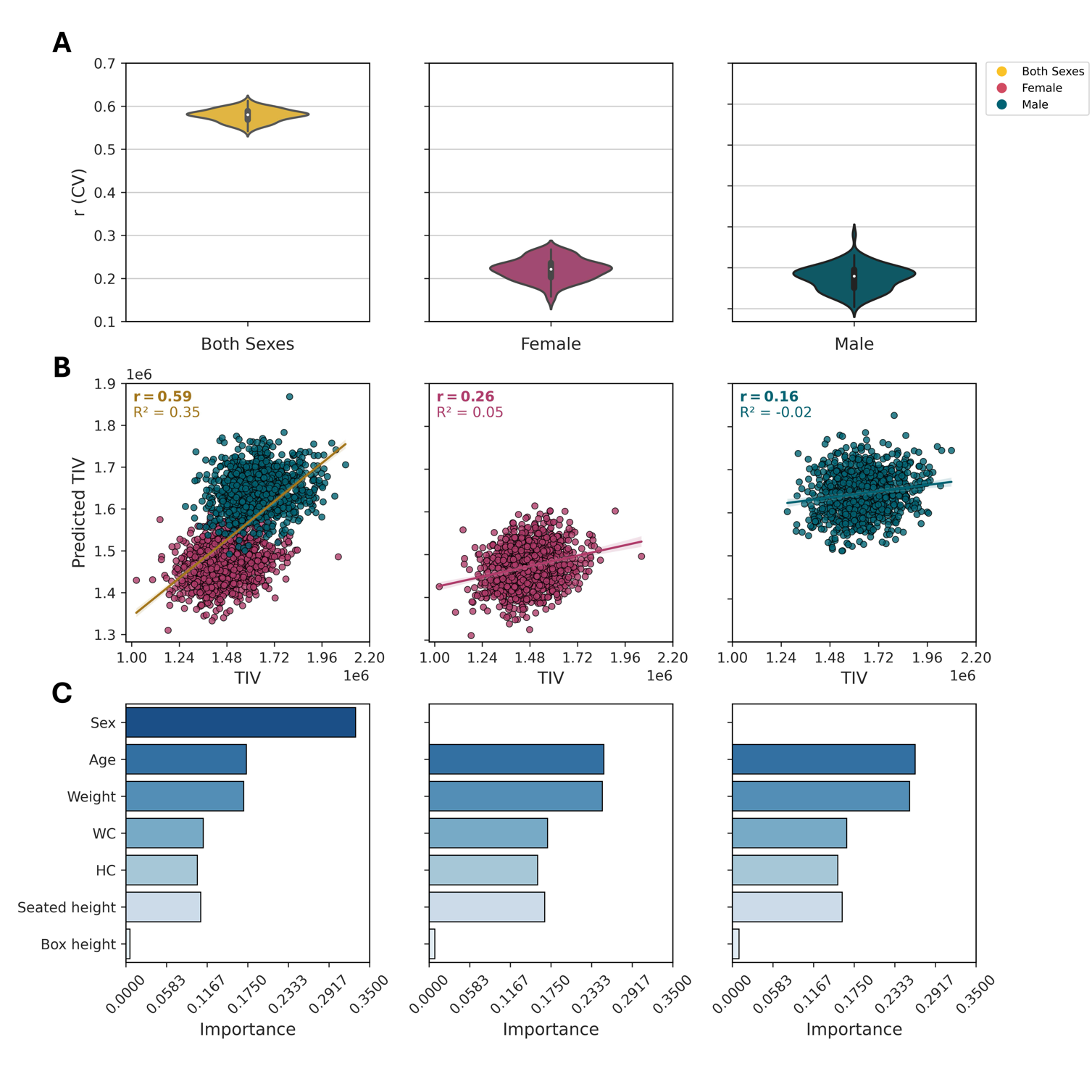


**Figure S4:** Prediction of brain size: Total brain volume (TBV) on CAT data using RF.


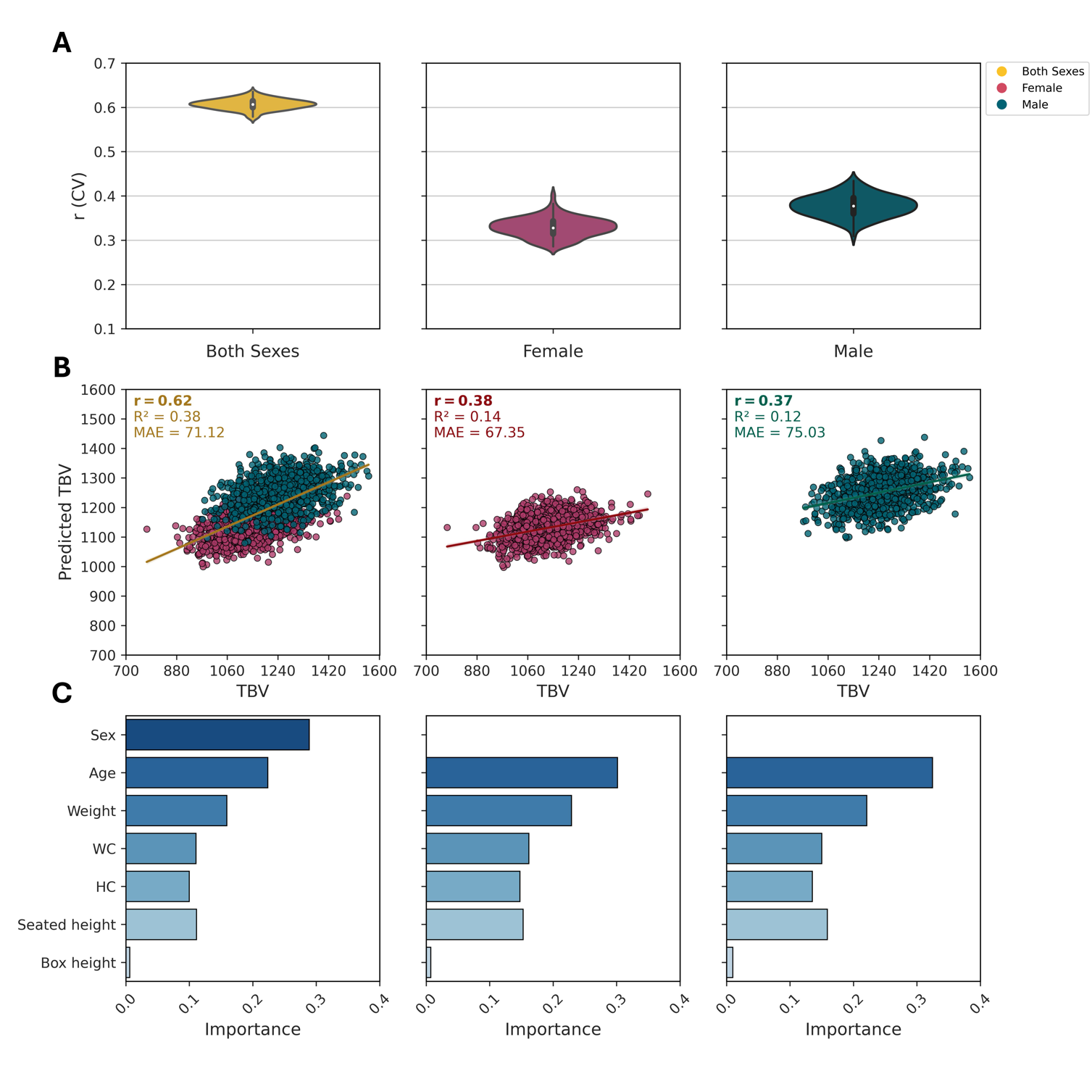


**Figure S5:** Prediction of brain size: Total brain volume (TBV) on FreeSurfer data using linear SVM


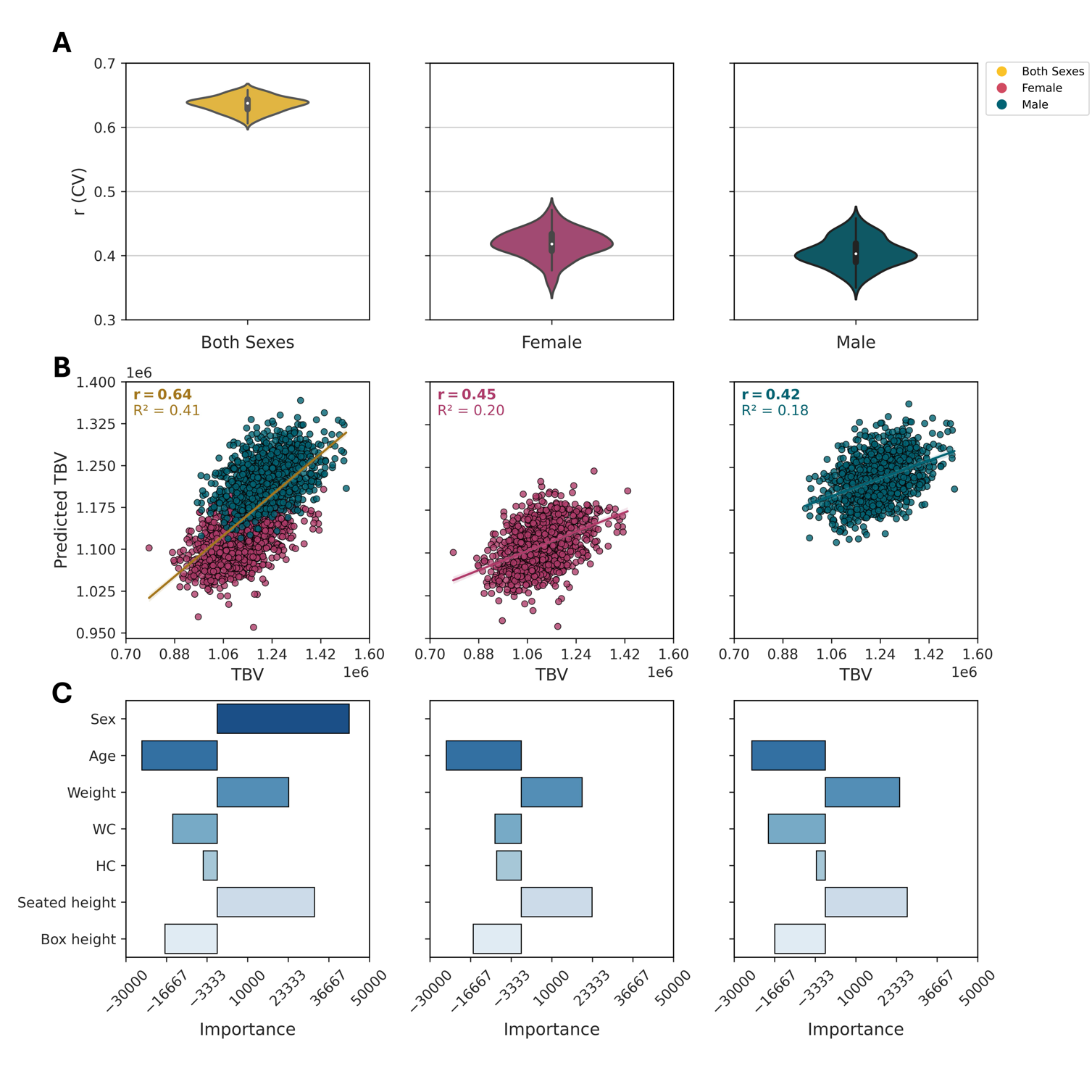


**Figure S6:** Prediction of brain size: Total brain volume (TBV) on FreeSurfer data using RF.


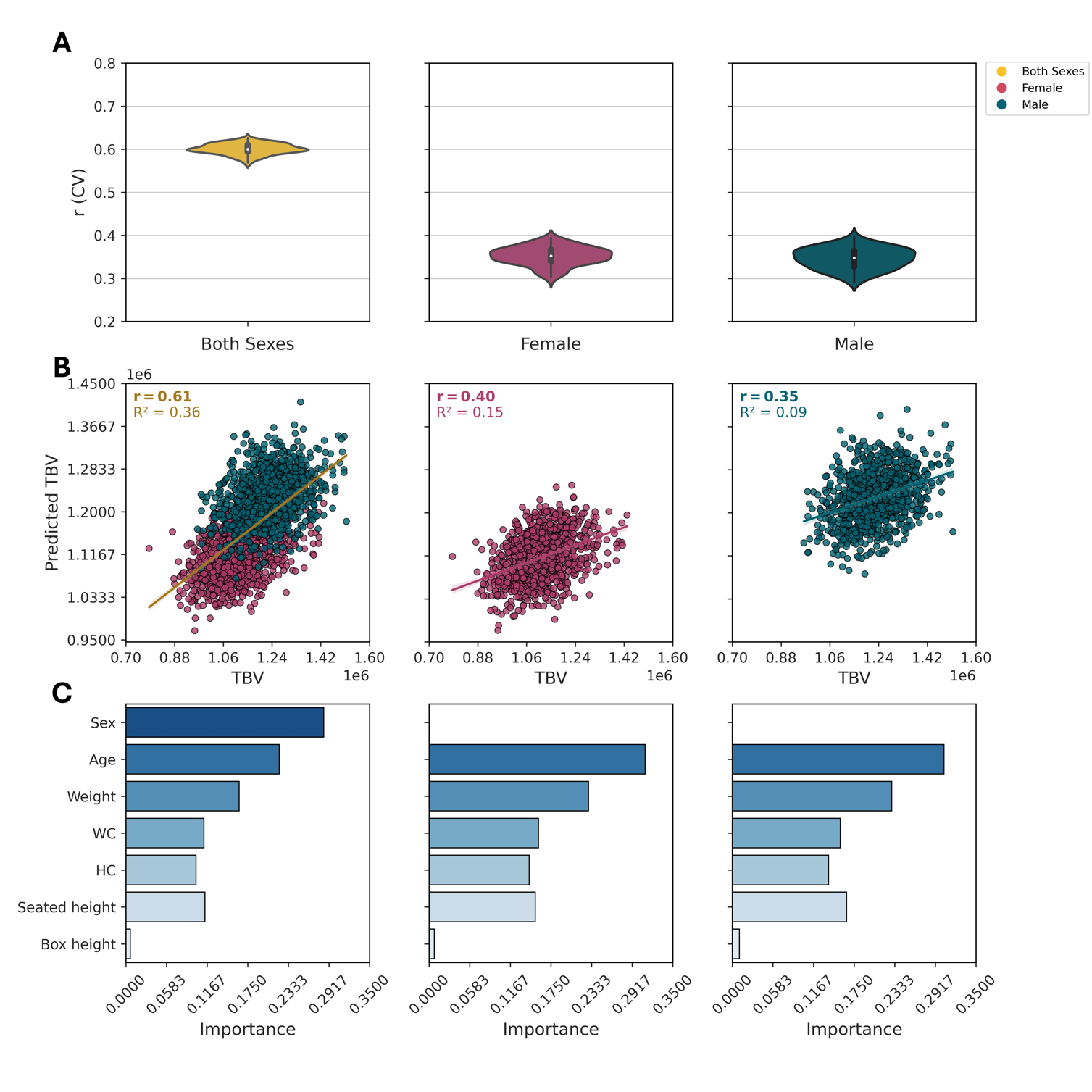


**Figure S7:** Impact of age on brain volumes for across-sex analysis on CAT data using RF.


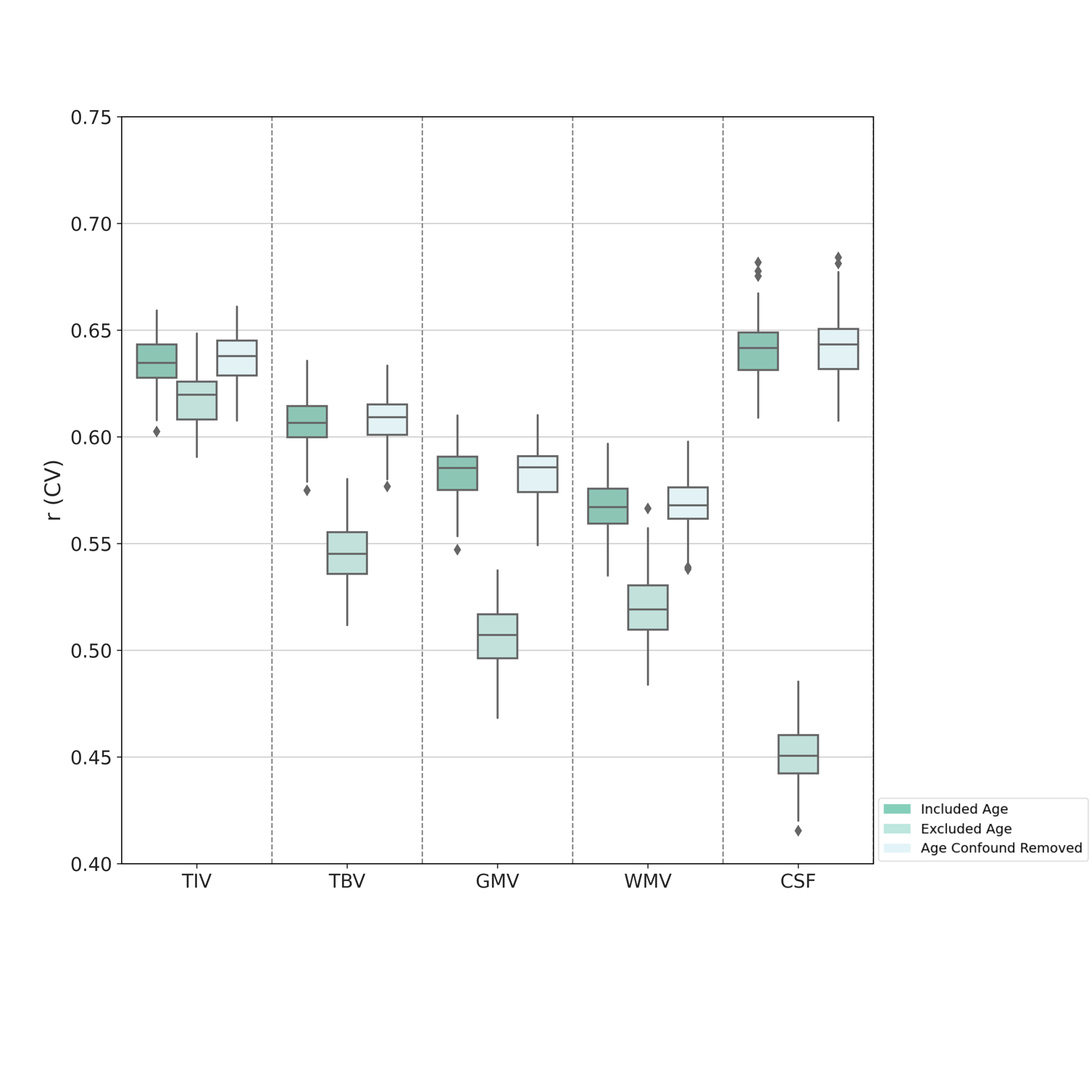


**Figure S8:** Impact of age on brain volumes for within-sex analysis on CAT data using linear SVM.


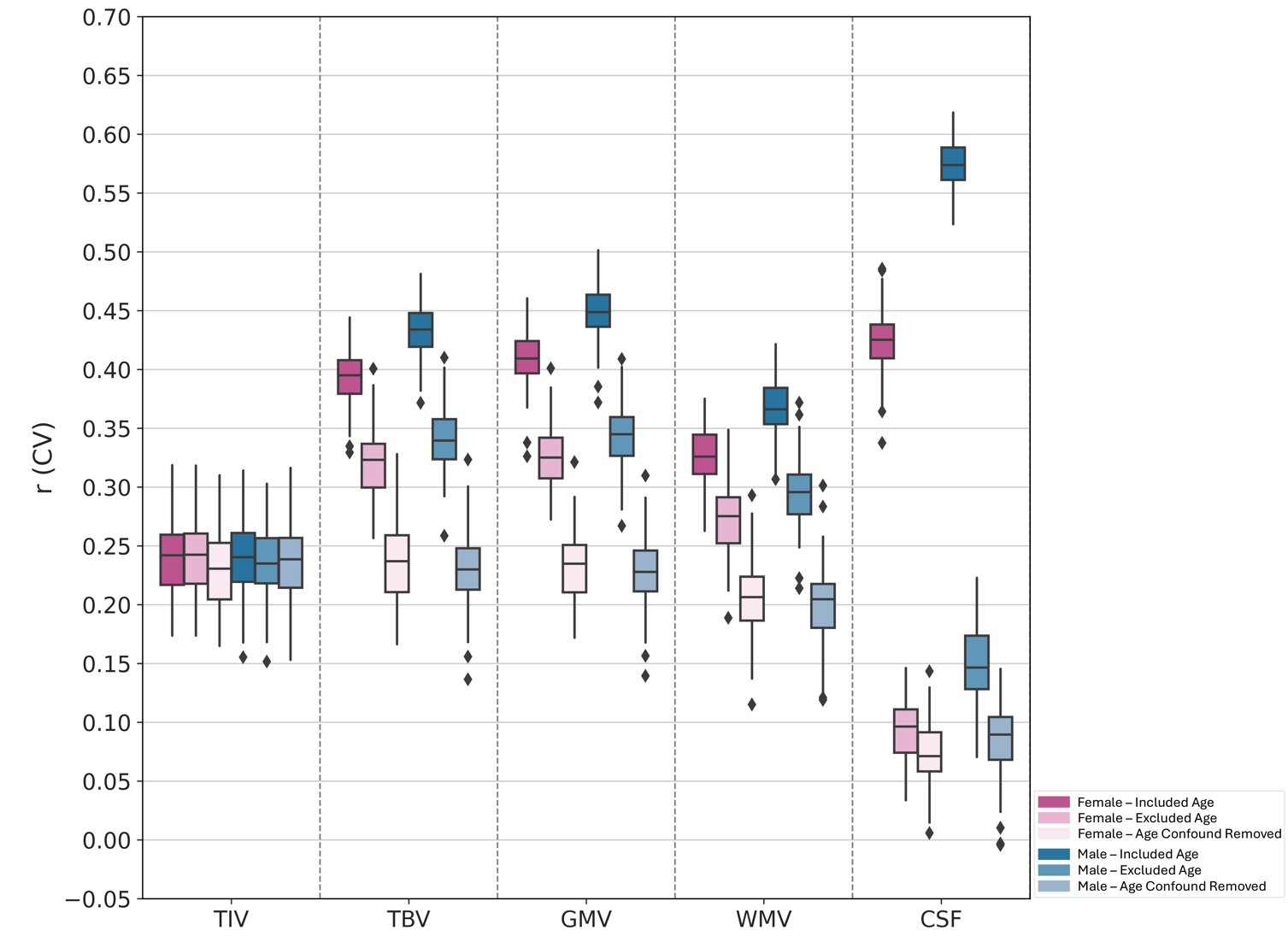


**Figure S9:** Impact of age on brain volumes for within-sex analysis on CAT data using random forest.


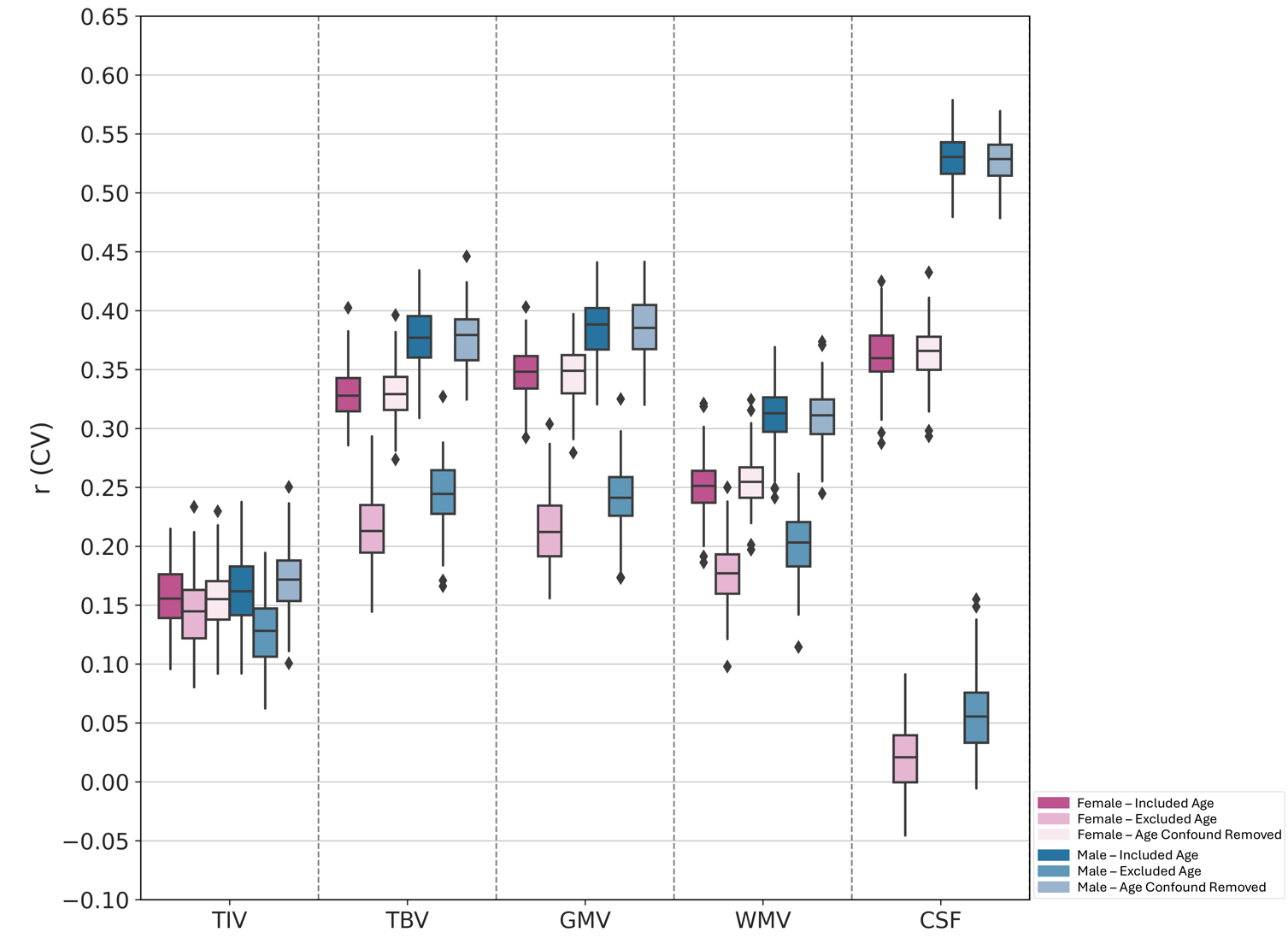


**Figure S10:** Impact of age on brain volumes for across-sex analysis on FreeSurfer data using linear SVM.


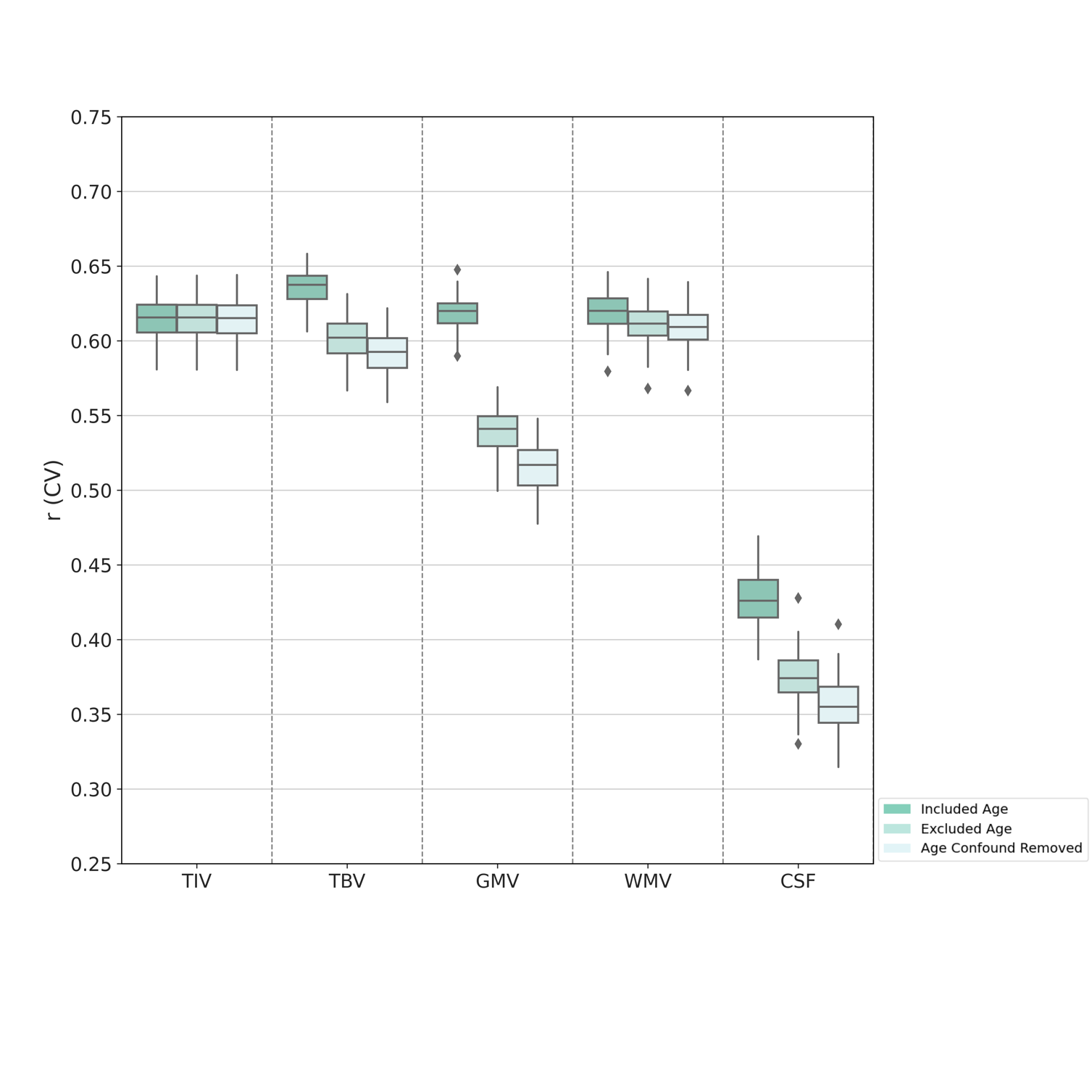


**Figure S11:** Impact of age on brain volumes for across-sex analysis on FreeSurfer data using RF.


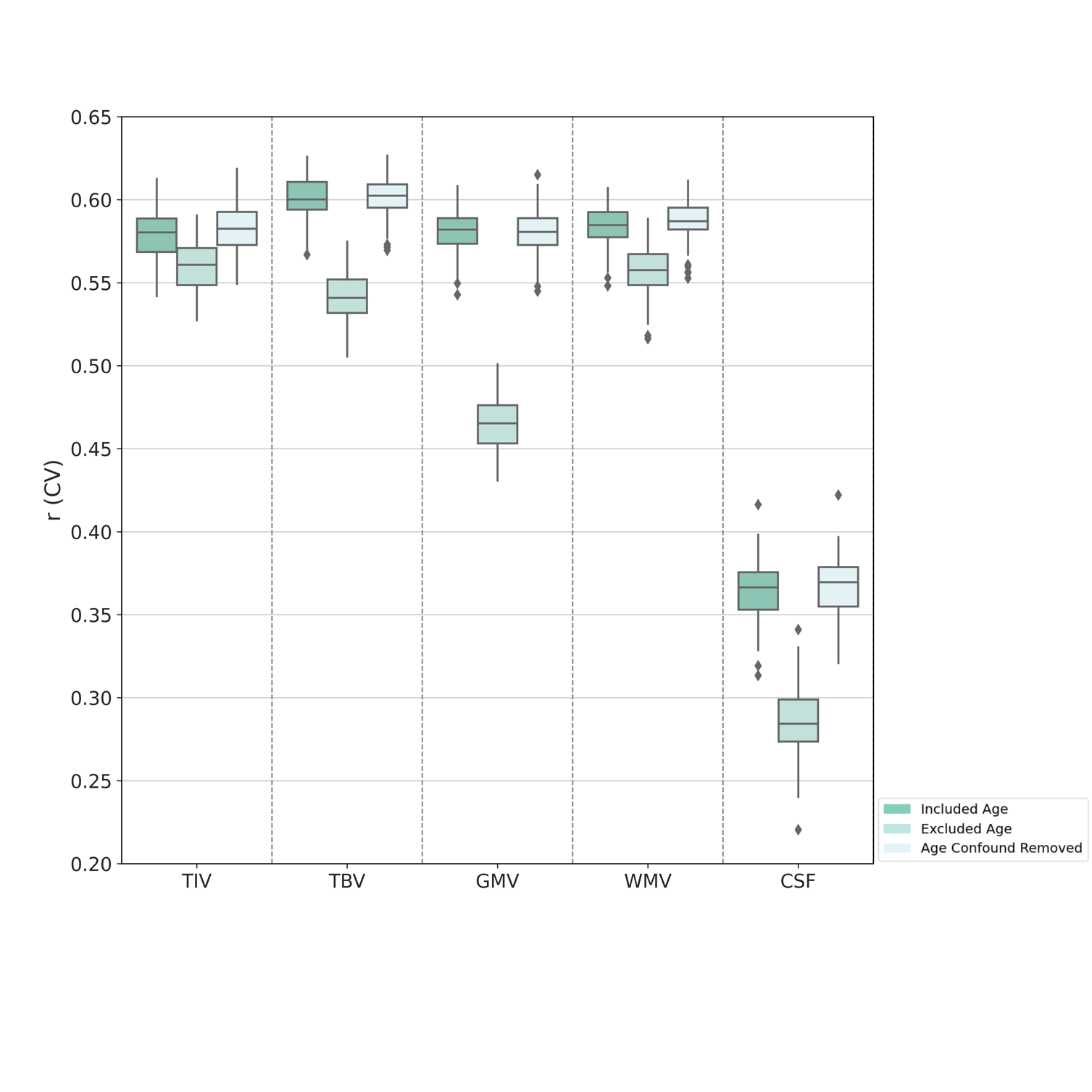


**Figure S12:** Impact of age on brain volumes for within-sex analysis on FreeSurfer data using linear SVM.


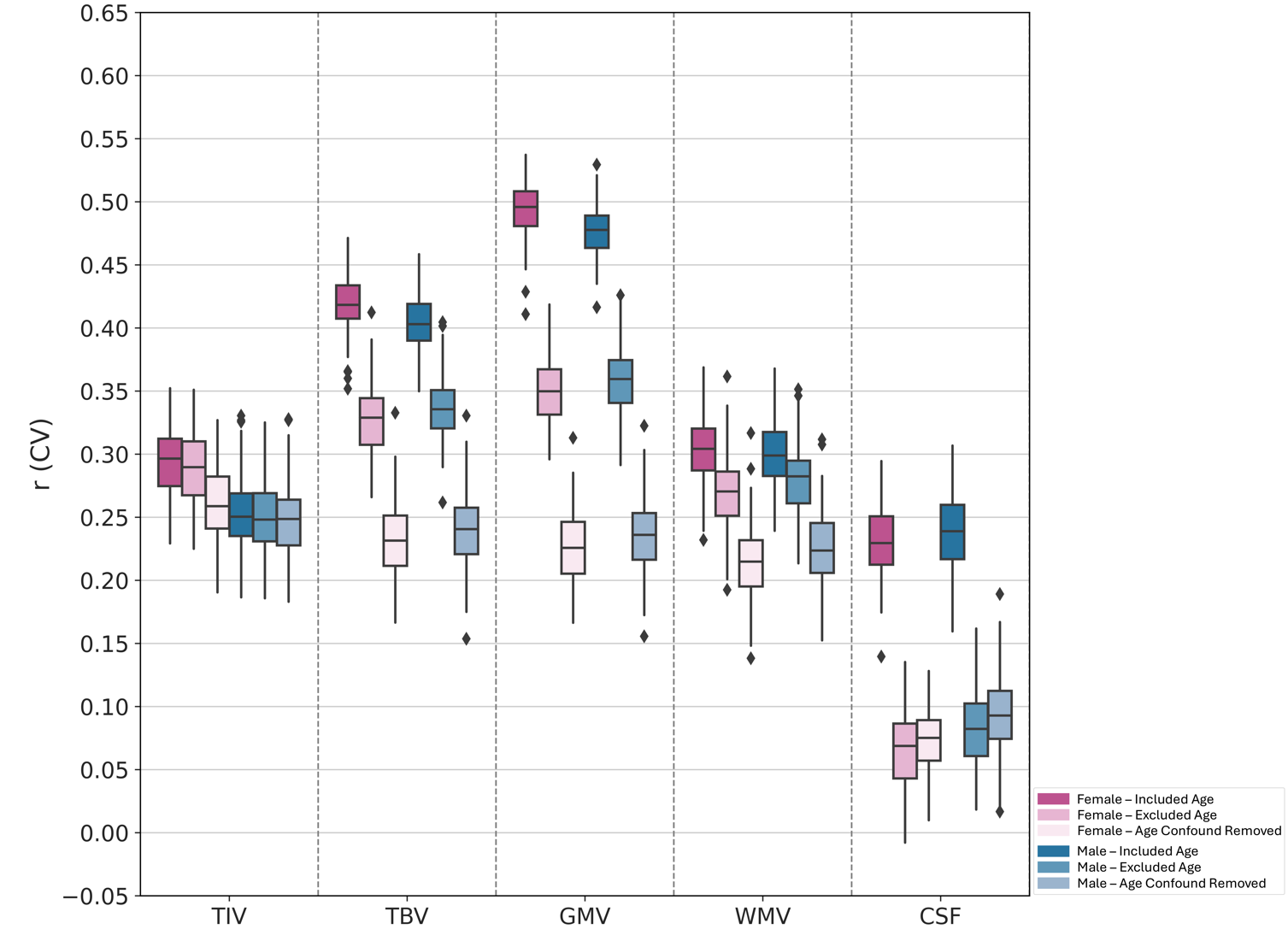


**Figure S13:** Impact of age on brain volumes for within-sex analysis on FreeSurfer data using RF.


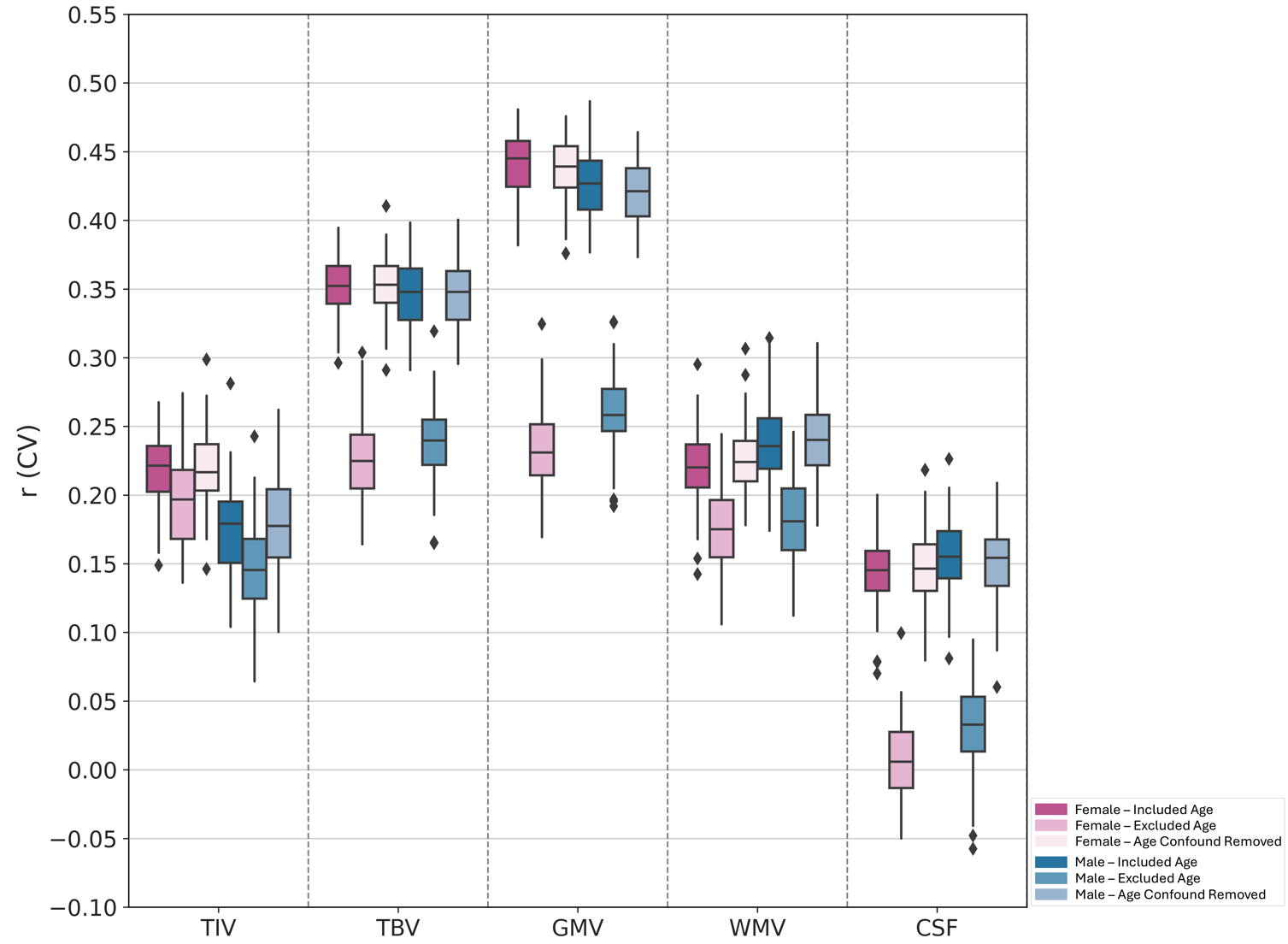

Supplement: Supplementary file 1 — Supplementary Material 1 [file 429_2025_3070_MOESM1_ESM.docx]
